# Prediction of Photosynthetic, Biophysical, and Biochemical Traits in Wheat Canopies to Reduce the Phenotyping Bottleneck

**DOI:** 10.3389/fpls.2022.828451

**Published:** 2022-04-11

**Authors:** Carlos A. Robles-Zazueta, Francisco Pinto, Gemma Molero, M. John Foulkes, Matthew P. Reynolds, Erik H. Murchie

**Affiliations:** ^1^Division of Plant and Crop Sciences, School of Biosciences, University of Nottingham, Leicestershire, United Kingdom; ^2^Global Wheat Program, International Maize and Wheat Improvement Center (CIMMYT), Texcoco, Mexico; ^3^KWS Momont Recherche, Mons-en-Pevele, France

**Keywords:** canopy photosynthesis, high-throughput phenotyping, PLSR, physiological breeding, RUE

## Abstract

To achieve food security, it is necessary to increase crop radiation use efficiency (RUE) and yield through the enhancement of canopy photosynthesis to increase the availability of assimilates for the grain, but its study in the field is constrained by low throughput and the lack of integrative measurements at canopy level. In this study, partial least squares regression (PLSR) was used with high-throughput phenotyping (HTP) data in spring wheat to build predictive models of photosynthetic, biophysical, and biochemical traits for the top, middle, and bottom layers of wheat canopies. The combined layer model predictions performed better than individual layer predictions with a significance as follows for photosynthesis *R*^2^ = 0.48, RMSE = 5.24 μmol m^–2^ s^–1^ and stomatal conductance: *R*^2^ = 0.36, RMSE = 0.14 mol m^–2^ s^–1^. The predictions of these traits from PLSR models upscaled to canopy level compared to field observations were statistically significant at initiation of booting (*R*^2^ = 0.3, *p* < 0.05; *R*^2^ = 0.29, *p* < 0.05) and at 7 days after anthesis (*R*^2^ = 0.15, *p* < 0.05; *R*^2^ = 0.65, *p* < 0.001). Using HTP allowed us to increase phenotyping capacity 30-fold compared to conventional phenotyping methods. This approach can be adapted to screen breeding progeny and genetic resources for RUE and to improve our understanding of wheat physiology by adding different layers of the canopy to physiological modeling.

## Introduction

Increasing crop biomass and radiation use efficiency (RUE; dry weight biomass produced per unit radiation intercepted) through the enhancement of photosynthesis has been presented as one of our best options to improve staple crop yields (Evans and Lawson, 2020). Multiple lines of evidence suggest that increased photosynthesis would stimulate higher yields, and moreover there is room for improvement within the existing crop systems (Zhu et al., 2010; Slattery et al., 2013; Kromdijk et al., 2016; South et al., 2019; Ainsworth and Long, 2021).

Most of the yield gains achieved in wheat (*Triticum aestivum* L.) from the Green Revolution came through the provision of the necessary resources for crop growth (i.e., water, nutrients, and pest control) and the introduction of *Rht* genes to increase harvest index (HI; proportion of biomass allocated in the grains) and plant structural integrity, thereby making it more responsive to irrigation and nutrients while reducing the risk of lodging (Reynolds et al., 2012). Currently, prebreeding efforts in wheat are focused on improving traits, such as aboveground biomass, light interception, HI, and RUE (Molero et al., 2019). Some of these traits are close to optimum, HI (close to 0.6), and light interception (canopies intercepting ∼95% of light), whereas RUE and biomass have a high potential for improvement. Therefore, increasing wheat photosynthesis has become a primary goal to increase yield (Murchie et al., 2009).

Biomass and RUE have increased in some wheat lines serendipitously without direct selection of RUE or photosynthetic traits. It has been suggested that RUE improvements in wheat need to be addressed through changes in leaf or spike photosynthesis (Carmo-Silva et al., 2017; Molero and Reynolds, 2020; Sanchez-Bragado et al., 2020), as previous studies have found a significant relationship between genetic variation in flag leaf light-saturated photosynthesis rates (A_*sat*_) and stomatal conductance (*gs*) with yield (Fischer et al., 1998; Gutiérrez-Rodrìguez et al., 2000; Reynolds et al., 2000; Gaju et al., 2016) and biomass (Reynolds et al., 2000; Gaju et al., 2016) at both pre- and postanthesis stages. However, recent studies have failed to find these correlations of single leaf photosynthesis or *gs* with yield (Driever et al., 2014; Silva-Pérez et al., 2020). To fully exploit genetic variation in existing germplasm, we need to not only develop high-throughput plant phenotyping (HTP) methods for faster assessment of photosynthetic-related traits but also find ways in which measurements of leaf or canopy photosynthesis will meaningfully correlate with canopy biomass and RUE to accelerate genetic yield gains.

Photosynthesis field research in wheat has been relatively slow in comparison to the study of other traits, such as aboveground biomass accumulation, light interception, RUE, and leaf and canopy pigment content, despite the latter requiring heavy manual labor in the field. This is a consequence of several factors that hinder accurate and representative estimations of photosynthetic traits under field conditions, which are mostly related to the complexity of photosynthesis as a trait. These include the time it takes to measure a leaf in the field for maximum assimilation rate under light saturating conditions (A_*sat*_, ∼15–25 min); the impracticality and low throughput techniques for measuring more complex photosynthetic traits, such as induction, CO_2_, or light concentration curves (A/C_*i*_, A/Q curves), and the confounding effect of crop phenology. Moreover, photosynthesis is typically measured in flag leaves which are usually exposed to light saturating conditions for most of the day, thus not representing the environmental conditions found across the whole canopy (Murchie et al., 2018).

Photosynthesis research gained a lot of interest after the seminal work from Farquhar et al. (1980). Since then methodologies were developed to measure, upscale to canopy level (“big leaf” models), and model photosynthesis considering mainly sunlit leaves, assuming that its rates would change with light intensity, penetration and distribution, N content, and leaf angles (Farquhar, 1989), with this modeling approach being applied in natural ecosystems (De Pury and Farquhar, 1997) and C_3_ and C_4_ crop systems by upscaling information from individual leaves to canopy level (Yin and Struik, 2009; Wu et al., 2019). Given that the prediction of canopy photosynthesis is improved with knowledge of photosynthesis at multiple canopy leaf layers, methodologies emerged to increase the spatio-temporal scales over which measurements can be made. Photosynthetic reactions can now be measured at cellular, leaf, and plant level with low to medium throughput phenotyping techniques (Murchie et al., 2018), and at ecosystem scale using sensors mounted on micrometerological stations (Baldocchi, 2003), and biome photosynthesis using chlorophyll fluorescence information collected from satellite sensors as a proxy of productivity can be estimated (Farquhar et al., 2001; Parazoo et al., 2014; Duveiller and Cescatti, 2016; Zhang et al., 2016). Although these are exciting methodologies used for photosynthesis research, the latter examples are not easy to deploy in wheat breeding programs as hundreds of lines are grown in plots placed next to each other, and upscaling information from leaves to plots can be hard due to the spatial scale mismatch in these methods which can vary from mm^2^ to km^2^.

There have been various investigations to assess photosynthetic-related traits at multiple canopy levels, such as A_*sat*_, RUE, the fraction of absorbed photosynthetically active radiation (fAPAR), maximum velocity of Rubisco carboxylation (V_*cmax*_), electron transport rate (J_*max*_), non-photochemical quenching (NPQ), and other chlorophyll fluorescence parameters, have been assessed in glasshouse studies coupled with 3D reconstructions using ray-tracing modeling in wheat (Townsend et al., 2018), rice (Burgess et al., 2016; Foo et al., 2020), maize (Cabrera-Bosquet et al., 2016), pearl millet, bambara groundnut (Burgess et al., 2017), and arabidopsis (Retkute et al., 2015) in different canopy layers. Under field conditions, A_*sat*_ measurements have been made with a custom made sensor (OCTOflux) which allowed the user to increase the phenotyping capabilities ∼4–7 times compared to conventional IRGAs (infrared gas analyzer) by measuring eight leaves at a time (Salter et al., 2018), A_*sat*_ measurements made in the top and bottom layers of wheat canopies (Salter et al., 2020), modeling with light response curves coupled with eddy covariance flux estimations of gross primary productivity (GPP; Hoyaux et al., 2008), and through image spectroscopy used to measure photochemical efficiency in wheat and maize (Pinto et al., 2016).

While these studies have shown that it is possible to estimate canopy photosynthesis through modeling, it has usually required laborious and complex manual measurements. Some have been used only in controlled environmental conditions or have not been tested in an HTP context limiting their use for physiological breeding. Additionally, these techniques are hard to deploy in the field, especially in breeding programs where hundreds of plots are grown in close proximity with limited space to maneuver large phenotyping equipment.

Recently, optical remote sensing techniques have gained attention due to the possibility of measuring hundreds or thousands of lines without the need of destructive sampling and in a small fraction of time compared to conventional phenotyping methods. Spectral data collected in the field has been used to calculate spectral indices or the full reflectance signature of an area of the electromagnetic spectrum, usually ranging from 350 to 2,500 nm to predict physiological traits at leaf or canopy scales (Ollinger, 2011; Gamon et al., 2019; Robles-Zazueta et al., 2021). Among the methods using the full spectral range, partial least squares regression (PLSR) modeling has become the gold standard for HTP modeling of physiological traits, such as leaf A_*sat*_; V_*cmax*_; J_*max*_; dark respiration; leaf osmotic potential; leaf C, N, and chlorophyll content; protein; phenols; sugars; leaf mass area; and specific leaf area (Serbin et al., 2012; Silva-Perez et al., 2018; Coast et al., 2019; Cotrozzi and Couture, 2020; Burnett et al., 2021; Furbank et al., 2021). Even though using hyperspectral reflectance data to predict physiological traits is not novel, the use of the spectra to predict photosynthetic, biophysical, and biochemical traits along different leaf layers has not been explored so far.

Our hypothesis is that models derived from rapid measurements of multiple layers of the canopy will produce better predictions than models created with individual leaf layers due to the unknown trait variability caused by a gradient from top to bottom of the canopy. The objectives of this study are to predict photosynthetic, biophysical, and biochemical traits using PLSR modeling, to compare the measurements of A_*sat*_ and *gs* with PLSR predictions, and to explore the use of these predictions as means to select wheat genotypes for higher RUE.

## Materials and Methods

### Plant Material and Experimental Design

Spring bread, wheat cultivars chosen from the Photosynthesis Respiration Tails (PS Tails) panel from the International Maize and Wheat Improvement Center (CIMMYT) were grown at CIMMYT’s Campo Experimental Norman E. Borlaug (CENEB) field station in Ciudad Obregon, Sonora, Mexico (27°23′46′′N, 109°55′42′′W, 38 mamsl) during the spring wheat growth season that encompasses early December–early May.

A subset of eight cultivars and advanced lines were studied in year 1 (Y1) and three more lines were added at years 2 and 3 (Y2 and Y3) to have a total of 11 lines. Germplasm from this panel is characterized by contrasting RUE expression at vegetative and grain filling stages, high aboveground biomass, and these lines are used for their promising high yield potential.

The experimental design was a randomized complete block design with three replicates in raised beds and two beds per plot (Y1) with the same experimental design, but four replications per genotype in Y2 and Y3. Sowing dates were December 5, 2017, December 6, 2018, and December 18, 2019 for Y1, Y2, and Y3, respectively. Emergence dates were December 12, 2017, December 12, 2018, and December 26, 2019 (Y1, Y2, and Y3, respectively). Harvest dates were May 8, 2018, April 30, 2019, and May 13, 2020 (Y1, Y2, and Y3, respectively). Seed rate was ∼250 seeds m^–2^ in 3 years. Irrigation was applied four times during the crop cycle in approximate 25-day intervals (pre-sowing, 25, 50, 75, and 100 days after emergence). Plants were grown under optimal conditions in the field with pests, weed control, and fertilization to avoid limitations to yield. In Y1 fertilization was applied in the form of urea (200 kg N ha^–1^) 25 days after emergence (DAE). For Y2, fertilization was divided into 100 kg N ha^–1^ 25 DAE and another 100 kg N ha^–1^ 58 DAE. Finally, for Y3 100 kg N ha^–1^ was applied 30 DAE and 50 kg N ha^–1^ 50 DAE; 50 kg P ha^–1^ was applied in the three cycles when the first application of N was made.

Phenology was scored according to the Zadoks growth scale for cereals (Zadoks et al., 1974). The growth stages recorded were initiation of booting (GS41, InB), anthesis (GS65, A), and physiological maturity (GS87, PM) when 50% of the shoots in the plot reached a particular stage. Meteorological data from a nearby station to the experimental site were collected for the whole crop cycle, and accumulated PAR was calculated for the growth stages where biomass was collected.

### Aboveground Biomass and Biophysical Traits

Aboveground biomass was sampled following Robles-Zazueta et al. (2021). Samples of biomass at InB, 7 days after anthesis (A7) and PM were collected. Biomass harvests were made in 0.4 m^2^ (40 days after emergence) and 0.8 m^2^ (InB, A7), leaving 25 and 50 cm, respectively, at the northern side of the plots to reduce border effects in subsequent biomass samplings. All fresh biomass was weighed, and a subsample of 50 shoots was weighed and dried in an oven at 70°C for 48 h to record dry weight. For biomass at PM, calculations were made from the measurement of yield components. For every growth stage, the aboveground biomass was calculated according to Pask et al. (2013):


(1) $$\text{Aboveground}\text{biomass}=\text{Subsample}\text{DW}\times \frac{\text{Total}\text{FW}\times \mathrm{Harvested}\mathrm{area}}{\mathrm{Subsample}\mathrm{FW}}$$

At InB and A7, 12 shoots were randomly selected for biomass partitioning. In the lab, plant organs were separated into stems, flag, second, third, and remaining leaves. After partitioning, leaf areas were measured using an area meter (LI 3100C, Licor Biosciences, Lincoln, NE, United States). Finally, samples were dried in an oven for 2 days at 70°C, weighted, and the data was used to calculate the leaf area index (LAI), specific leaf area (SLA), and leaf mass area (LMA) as follows:


(2) $$\text{LAI}=\frac{\mathrm{Green}\mathrm{leaf}\mathrm{lamina}\mathrm{area}}{\#\mathrm{stems}m^{2}}$$

(3) $$\text{SLA}=\frac{\mathrm{Leaf}\mathrm{green}\mathrm{area}}{\mathrm{Leaf}\mathrm{dry}\mathrm{mass}}$$

(4) $$\mathrm{LMA}=\frac{\mathrm{Leaf}\mathrm{dry}\mathrm{mass}}{\mathrm{Leaf}\mathrm{green}\mathrm{area}}$$

### Radiation Use Efficiency

Radiation use efficiency was estimated from the slope of the linear regression between accumulated aboveground biomass and the corresponding accumulated intercepted PAR during the determined growth period (Monteith, 1977). Incoming radiation from a nearby meteorological station was used to estimate the accumulated PAR multiplying irradiance by a factor of 0.45 to convert it to PAR, and ceptometer (AccuPAR LP-80, Decagon, Pullman, WA, United States) readings were used to correct the accumulated radiation for the fraction of absorbed PAR by each genotype following the same procedure presented in Robles-Zazueta et al. (2021).

### Photosynthesis and Chlorophyll Measurements

Spot measurements of A_*sat*_, *gs*, the maximum efficiency of PSII photochemistry under light conditions (Fv′/Fm′), and PSII quantum yield (ΦPSII) were made using an IRGA (Licor 6400 XT, Licor Biosciences, Lincoln, NE, United States) at InB (Y1 and Y2) and A7 (Y1, Y2, and Y3) coupled with the leaf chamber fluorometer (6400-40 Licor Biosciences, Lincoln, NE, United States). Photosynthetic measurements were made at the flag (top of the canopy), second (middle of the canopy), and third (bottom of the canopy) leaves in two healthy shoots per plot with light conditions set at 1,800 μmol m^–2^ s^–1^ PAR, which are light saturating conditions in our study site, and the leaves were acclimated for ∼15–20 min until steady state was reached. Chlorophyll content was measured using a SPAD-502 meter (Konika Minolta, Tokyo, Japan) in the same leaves where photosynthesis was measured (Pask et al., 2013).

Measurements were performed between 10:00 and 15:00 as this timeframe has been found to maximize the stability and accuracy of the measurements (Evans and Santiago, 2014). Then CO_2_ assimilation (A_*sat*_) and stomatal conductance (*gs*) were upscaled from leaves to canopy level by multiplying each individual layer value by the LAI of its corresponding layer. This is an adaptation of the protocol for upscaling C and N content proposed by Gara et al. (2019). Calculations are shown in Eq. 5:


(5) $$\mathrm{Canopy}\mathrm{Photosynthesis}=\sum \left({\text{A}}_{\text{sat}}\mathrm{FL}x\mathrm{LAI}\mathrm{FL}\right)+\left({\text{A}}_{\text{sat}}\mathrm{SL}x\mathrm{LAI}\mathrm{SL}\right)+\left({\text{A}}_{\text{sat}}\mathrm{TL}x\mathrm{LAI}\mathrm{TL}\right)$$

where A_*sat*_ is CO_2_ assimilation, LAI is leaf area index, and FL, SL, and TL are flag leaf, second leaf, and third leaf, respectively.

For *gs*, an average of the three layers of the canopy was estimated to obtain a *gs* pooled value of the canopy to assess if the average *gs* of any leaf in the canopy correlated better with the traits of interest.

### Total C and N Content

Flag, second, and third leaf samples from each genotype were collected from the field to obtain the total C and N content at GS41 and GS65 + 7 days in Y1 and Y2. Leaf samples were dried in an oven at 70°C for 48 h, then finely grounded, weighted, and analyzed with dry combustion Dumas method using an elemental analyzer (Flash 2000, Thermo Scientific, Waltham, MA, United States).

### Leaf Hyperspectral Reflectance

Hyperspectral reflectance was measured on the adaxial sides of the same leaves where gas exchange data were collected. Measurements were made using a leaf clip equipped with a halogen bulb light source (ASD Field Spec 3, Boulder, CO, United States). Reflectance was measured in the flag, second, and third leaves at the same growth stages as photosynthesis measurements between 10:00 and 15:00, making sure there were no water or dust particles in the leaves to avoid noisy readings.

### Statistical Analysis

Leaf spectral reflectance (350–2500 nm) collected at the three positions of the canopy was used to predict the photosynthetic, biophysical, and biochemical traits using PLSR (Serbin et al., 2012, 2014) with the orthogonal scores algorithm (*oscorespls*) from the R package pls (Mevik and Wehrens, 2007). Before constructing the models, outliers of the traits measured were removed (±3 σ) and the dataset was divided for training (70%) and validation (30%) randomly using the sample function from R Studio, which has an equal probability of selecting any numeric vector within a dataset (RStudio Team, 2020). This procedure is characterized by reducing the risk of model overfitting, as shown in previous studies, that measured photosynthetic and physicochemical traits in leaves (Serbin et al., 2012, 2014).

A jackknife resampling test with 1,000 iterations was done to estimate the variance and model bias. Jackknife resampling is also known as leave-one-out cross-validation, which implies that for any dataset of size *n*, the estimation of a given parameter in the dataset will be done by the addition of the parameter estimates from a subsample of size *n* −1 (Jiang et al., 2002). Then the number of principal components used in the model was defined by the smallest root mean square error from the cross-validation stage (RMSEP CV) in conjunction with the smallest prediction of the residual sum squares (PRESS) from the training model according to Serbin et al. (2014). After the validation process, regression coefficients and intercepts were generated and multiplied by the reflectance value of each individual wavelength to predict the abovementioned traits (Serbin et al., 2014; Silva-Perez et al., 2018).

The models were built based on two approaches: individual layers and all canopy layers combined. The size of the training and validation dataset and statistical parameters used to evaluate the models is presented in Table 1. Then results were compared to define which approach was better to predict the physiological traits based on the regression coefficient (*R*^2^), root mean square error (RMSE), and the model bias (Table 1). Furthermore, variable importance in projection (VIP) scores for each physiological trait were calculated to define which areas of the electromagnetic spectrum carry significant weight for the model construction, where values >1 represent areas of higher importance compared to values <1.

**TABLE 1:** Statistical parameters used to build the partial least squares regression (PLSR) models.

Trait	Layer	N T	N V	RMSEP CV (Trait units)	N Comp	*R*^2^ T	*R*^2^ V	RMSE_V (Trait units)	Bias_V (%)
A_sat_	Top	157	69	4.47	10	0.23	0.11	4.96	–0.78
	Middle	155	67	4.51	11	0.48	0.34	6.6	1.28
	Bottom	146	64	4.65	10	0.16	0.07	5.82	0.54
	Combined	525	198	5.19	15	0.46	0.48	5.24	–0.32
*gs*	Top	155	67	0.14	5	0.11	0.17	0.16	0.004
	Middle	149	64	0.14	5	0.29	0.37	0.15	–0.01
	Bottom	155	69	0.14	5	0.28	0.22	0.15	0.019
	Combined	460	199	0.14	13	0.34	0.36	0.14	0.005
Fv′/Fm′	Top	151	66	0.03	12	0.27	0.36	0.04	0.008
	Middle	154	67	0.03	10	0.03	0.18	0.81	–0.81
	Bottom	152	67	0.04	6	0	0.1	0.05	–0.13
	Combined	458	199	0.04	14	0.16	0.17	0.05	–0.003
ΦPSII	Top	157	69	0.03	12	0.49	0.29	0.04	0.0037
	Middle	154	64	0.04	8	0.43	0.52	0.04	0.001
	Bottom	145	63	0.03	12	0.26	0.56	0.04	–0.004
	Combined	458	198	0.04	14	0.57	0.57	0.04	–0.003
SPAD	Top	150	66	2.08	5	0.61	0.63	2.2	–0.03
	Middle	155	68	1.99	10	0.24	0.24	1.56	0.54
	Bottom	152	69	2.88	3	0.07	0.04	2.79	–0.25
	Combined	460	198	2.4	13	0.47	0.48	2.48	0.217
Total C	Top	89	40	1.3	8	0.38	0.3	1.9	–0.922
	Middle	85	39	1.71	1	0.05	0.03	1.86	–0.48
	Bottom	84	37	1.73	3	0.06	0.02	1.95	0.175
	Combined	260	114	0.66	27	0.33	0.35	1.5	0.15
Total N	Top	90	40	0.11	20	0	0	0.62	0.093
	Middle	88	39	0.38	6	0.35	0.3	0.56	0.0714
	Bottom	87	38	0.38	9	0.44	0.31	0.53	0.0082
	Combined	266	116	0.44	8	0.3	0.38	0.49	0.008
SLA	Top	153	67	3.74	3	0.03	0.17	4.18	0.033
	Middle	122	54	2.92	10	0.11	0.01	3.66	0.354
	Bottom	136	59	3.07	13	0.57	0.63	4.38	–0.241
	Combined	413	178	4.38	6	0.31	0.32	5.23	–0.117
LMA	Top	150	65	0.01	3	0.07	0.05	0.01	–0.0013
	Middle	119	52	0.01	2	0.01	0.01	0.01	–0.002
	Bottom	134	60	0	14	0.49	0.56	0.01	–0.00008
	Combined	450	195	0.01	14	0.49	0.46	0.01	–0.0013

*The lowest RMSEP CV was used to select the ideal number of components. NT, datapoints used for the training dataset; NV, datapoints used for validation dataset; RMSEP CV, root mean square error from cross-validation; N Comp, number of components; R^2^ T, determination coefficient from test model; R^2^ V, determination coefficient from validation model; RMSE_V, root mean square error from validation; Bias_V, validation model bias.*

Bilinear unbiased estimators (BLUEs) were calculated for each trait measured on the field using the general linear mixed model with META-R v 6.04 (Alvarado et al., 2020). Physiological traits were adjusted using the days to InB as a covariate for traits measured during the vegetative stage and days to A for traits measured during the grain filling stage when no significant statistical differences were found. For the analysis combined across the 3 years, the following model was used:


(6) $$Y_{ijkl}=\mu +Env_{i}+Rep_{j}\left(Env_{i}\right)+Gen_{l}+Env_{i}xGen_{l}+Cov+\varepsilon_{ijkl}$$

Where Y*_*ijkl*_* is the trait of interest, μ is the mean effect, Env*_*i*_* is the effect of the *i*th environment, Rep*_*j*_* is the effect of the *j*th replicate, Gen*_*l*_* is the effect of the *l*th genotype, Env*_*i*_* × Gen*_*l*_* are the effects of the *i*th environment and the environment × genotype interaction, Cov is the effect of the covariate, and ε*_*ijkl*_* is the error associated with the environment *i*, replication *j*, *k*th incomplete block, and *l*th genotype. All the effects in the model are random, with exception of genotype and covariate which are fixed. In this study, the term environment refers to the year where data was collected (Y1, Y2, or Y3), therefore three environments were analyzed.

Finally, to compare our estimations of A_*sat*_ and *gs* with the predictions from PLSR models, we used the equations generated from the validation models and calculated BLUEs of the predicted A_*sat*_, *gs*, and LAI to upscale these predictions to a canopy level.

## Results

### Canopy Layer Position and Phenological Effects on Photosynthetic Traits

Photosynthetic traits were greater in the middle leaf layer of the canopy than the top layer in InB, and strong statistical differences were found between the middle and bottom layers of the canopy, with greater A_*sat*_ and *gs* rates in the middle layer. With exception of total C content, statistically significant differences between layers were found in all the physiological traits measured in this study. Similarly, differences between growth stages were strongly significant for all the traits. This highlights the importance of considering adding data from different phenological stages to build more robust models that could predict these traits at any given point in time of the wheat-growing season (Table 2).

**TABLE 2:** Mean ± standard deviation values of physiological traits measured in the three field cycles.

	Initiation of booting			7 days after anthesis										
Trait	T	M	B	T	M	B	L	G × L	Env × L	G × Env × L	GS	L × GS	GS × Env	L × GS × Env
A_*sat*_	25.5 ± 3.07	26.9 ± 4.7	15.9 ± 4.12	22 ± 3.96	16.7 ± 3.98	10.1 ± 3.7	***	ns	ms	ns	***	***	ns	ns
*gs*	0.39 ± 0.12	0.46 ± 0.15	0.34 ± 0.14	0.32 ± 0.09	0.25 ± 0.09	0.15 ± 0.06	***	ns	ms	ns	***	***	ns	ns
Fv′/Fm′	0.47 ± 0.03	0.52 ± 0.03	0.5 ± 0.027	0.52 ± 0.03	0.54 ± 0.03	0.51 ± 0.03	***	ns	*	ns	***	***	***	ns
ΦPSII	0.31 ± 0.02	0.31 ± 0.037	0.26 ± 0.04	0.25 ± 0.029	0.22 ± 0.029	0.17 ± 0.03	***	ns	ns	ns	***	***	***	ns
LAI	1.53 ± 0.33	1.73 ± 0.33	1.75 ± 0.34	1.43 ± 0.3	2.31 ± 0.62	2.14 ± 0.54	***	ns	***	ns	***	***	***	***
C^†^	44.5 ± 1.49	44.2 ± 1.61	42.6 ± 1.64	40.4 ± 2.15	41.1 ± 4.16	43.4 ± 5.55	ns	ns	ms	ns	***	***	ns	**
N^†^	4.4 ± 0.34	4.6 ± 0.4	4.2 ± 0.47	4 ± 0.48	3.7 ± 0.64	3.6 ± 0.8	**	ns	ns	ns	***	ns	ns	**
SLA	19.8 ± 3	20.4 ± 2.15	24.2 ± 1.92	17.6 ± 2.27	37.4 ± 5.04	31.9 ± 3.83	***	ns	***	ns	***	***	***	***
LMA	0.05 ± 0.01	0.05 ± 0.005	0.04 ± 0.004	0.06 ± 0.01	0.04 ± 0.004	0.04 ± 0.005	***	ns	***	ns	***	***	***	***
SPAD	45.8 ± 2.16	48.1 ± 1.71	45.3 ± 2.34	50.6 ± 1.93	49.3 ± 2.18	44.4 ± 2.78	***	ns	*	ns	***	***	***	***

T, top layer of the canopy; M, middle layer of the canopy; B, bottom layer of the canopy. ***Significant at p < 0.001, **significant at p < 0.01, *significant at p < 0.05; ms, 0.1 > p > 0.05; ns, not significant.

*^†^Measured only in Y1 and Y2.*

*L, layer; G × L, genotype × layer; Env × L, environment × layer; G × Env × L, genotype × environment × layer; GS, growth stage; L × GS, layer × growth stage; GS × Env, growth stage × environment; L × GS × Env, layer × growth stage × environment.*

### Predicting Photosynthetic, Biophysical, and Biochemical Traits With Hyperspectral Reflectance

**FIGURE 1:**
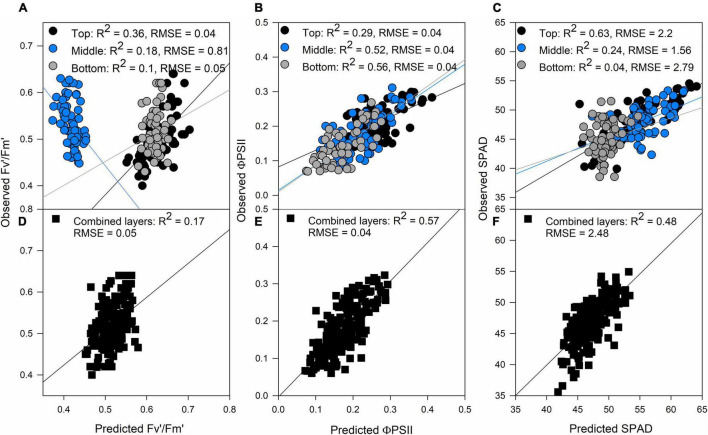
Validation results of partial least squares regression (PLSR) models predicting Fv′/Fm′ **(A)**, ΦPSII **(B)**, and SPAD **(C)** by separating each layer of the canopy (top panels) and predictions of Fv′/Fm′ **(D)**, ΦPSII **(E)**, and SPAD **(F)** combining all the layers of the canopy (black squares). Black dots: top of the canopy, blue dots: middle of the canopy, gray dots: bottom of the canopy. The lines represent the linear regression between predictions and ground truth data.

The photosynthetic trait with the smallest accuracy prediction of all, both for the separated (top: *R*^2^ = 0.36, middle: *R*^2^ = 0.18, bottom: *R*^2^ = 0.1) and combined layers approach (*R*^2^ = 0.17; Figures 1A,D, respectively) was Fv′/Fm′. On the other hand, ΦPSII and SPAD predictions were traits with a high correlation between observations and predictions with both approaches. In the case of ΦPSII, the middle and bottom layer of the canopy were crucial to improve model accuracy, while the top layer had a smaller correlation value compared to predictions made with the three layers (Figures 1B,E); this was opposite to SPAD predictions where the top layer of the canopy had the highest correlation, and therefore the most influence on model accuracy when combining the three layers of the canopy (Figures 1C,F). In the case of these three traits, we found that the two approaches produced similar correlations between observations and predictions, but in the case of Fv′/Fm′ the combined layer approach was better (Figure 1D).

**FIGURE 2:**
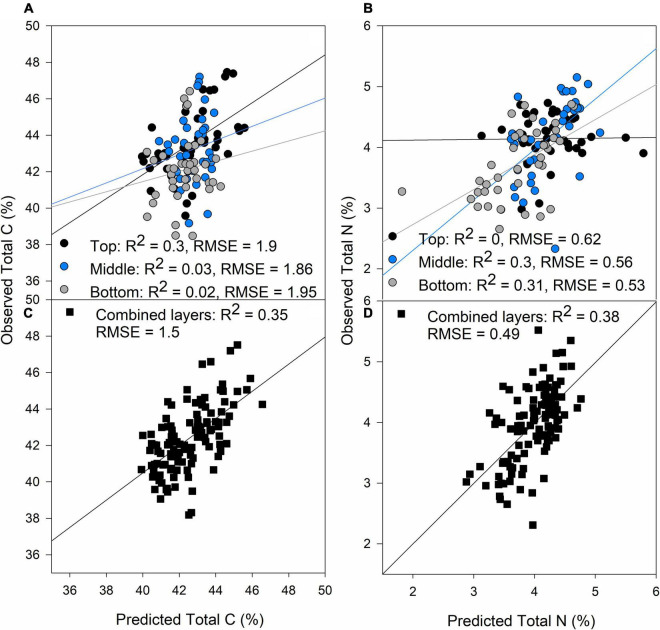
Validation results of PLSR models predicting Total C **(A)** and Total N **(B)** by separating each layer of the canopy (top panels) and predictions of Total C **(C)** and Total N **(D)** combining all the layers of the canopy (black squares). Black dots: top of the canopy, blue dots: middle of the canopy, gray dots: bottom of the canopy. The lines represent the linear regression between predictions and ground truth data.

Total C (%) and N (%) predictions were poor compared to the photosynthetic traits, possibly due to a smaller sampling size compared to the other traits predicted (Table 1) and the experimental conditions, where N was not a limiting factor coupled with low genetic variability as only eleven lines were studied, and this could have had an effect on the low predictions of these two traits. The top layer produced best predictions for C content (Figure 2A, *R*^2^ = 0.3, RMSE = 1.9), whereas the middle and bottom layers were more important for N content predictions (Figure 2B; *R*^2^ = 0.3, *p* < 0.001 and *R*^2^ = 0.31, *p* < 0.001, respectively). When all the layers were combined, predictions were better than separating the layers for both traits with RMSE of 1.5 and 0.49% for C and N prediction, respectively (Figures 2C,D, respectively). N content decreased from top to bottom of the canopy, but C content was equally distributed through the canopy (Table 2).

**FIGURE 3:**
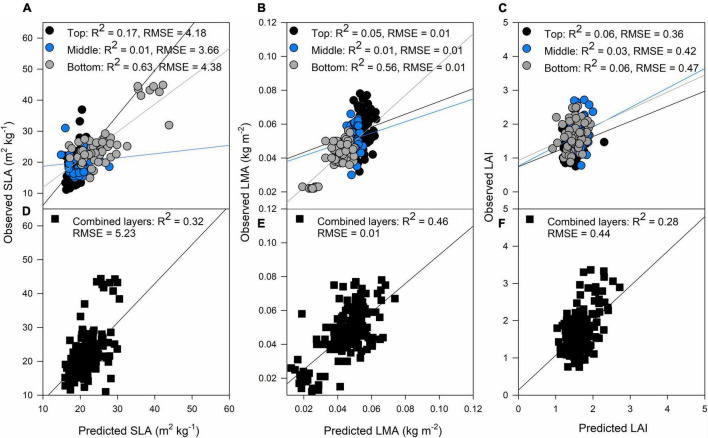
Validation results of PLSR models predicting specific leaf area (SLA) **(A)**, LMA **(B)**, and LAI **(C)** by separating each layer of the canopy (top panels) and predictions of SLA **(D)**, LMA **(E)**, and LAI **(F)** combining all the layers of the canopy (black squares). Black dots: top of the canopy, blue dots: middle of the canopy, gray dots: bottom of the canopy. The lines represent the linear regression between predictions and ground truth data.

The bottom layer predictions were more accurate than the top and middle layers for the biophysical traits. Predictions at the bottom for SLA were *R*^2^ = 0.63, *p* < 0.001 and for LMA were *R*^2^ = 0.56, *p* < 0.001 (Figures 3A,B, respectively). When combining the three layers, the results were similar for LMA, but in the case of SLA, the separated layer model produced better correlations between observations and predictions (Figures 3D,E). These results comply with our field observations as narrower smaller leaves at the top layer, and broader, larger leaves at the middle and bottom layers of the canopy were found (Table 2).

**FIGURE 4:**
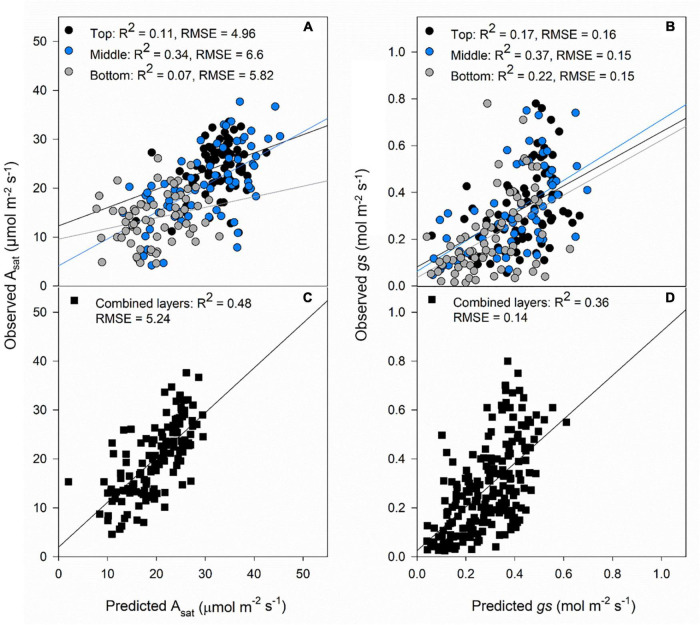
Validation results of PLSR models predicting A_*sat*_ **(A)** and *gs* **(B)** by separating each layer of the canopy (top panels) and predictions of A_*sat*_ **(C)** and *gs* **(D)** combining all the layers of the canopy (black squares). Black dots: top of the canopy, blue dots: middle of the canopy, gray dots: bottom of the canopy. The lines represent the linear regression between predictions and ground truth data.

**FIGURE 5:**
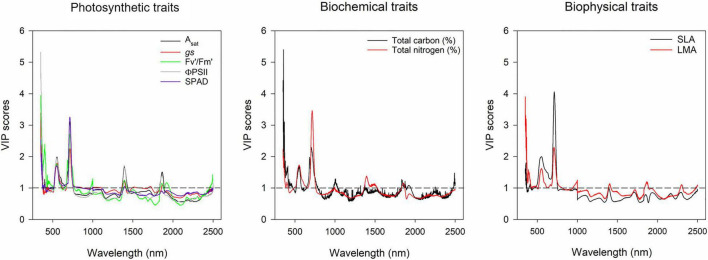
Variable importance in projection plot of gas exchange, biochemical and biophysical traits of the models built with all the canopy layers combined. *Y*-axis represents the variance importance score where values >1 represent wavelengths with greater importance for the model predictions. *X*-axis represents the wavelength (nm). Physiological traits are represented by different colors in each plot as indicated in the figure legends.

The results from the models using canopy layers separated and combined are presented in this section. Our results indicate that photosynthetic traits prediction was better using the combined approach rather than the separated (Figure 4). A_*sat*_ predictions from the combined model had RMSE of 5.24 μmol m^–2^ s^–1^ (Figure 4C), and *gs* RMSE of 0.14 mol m^–2^ s^–1^ (Figure 4D). For these two traits, the middle layer had more importance for model accuracy (Figures 4A,B). We recognize that our photosynthesis modelling results can increase by improving LAI predictions (Figures 3C,F). Variable importance in projection (VIP) scores were calculated to find spectrum areas, with the most importance for model building. We found three main areas with the greatest importance in the building of the photosynthetic, biophysical, and biochemical models at 350–369, 527–575, and 671–750 nm (Figure 5). After smaller peaks in the shortwave infrared region (SWIR), spectral wavelengths above 1,436 nm lacked importance for the predictive model building (VIP scores < 1; Figure 5).

### Photosynthetic Predictions and Their Relationship With Radiation Use Efficiency

**FIGURE 6:**
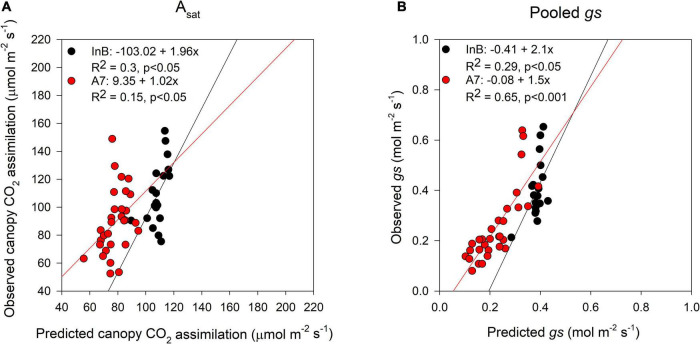
Comparison between predictions (*X*-axis) and ground truth data (*Y*-axis) of canopy assimilation **(A)** and stomatal conductance **(B)**. Black dots represent data from initiation of booting and red dots data from 7 days after anthesis. Data shown are the observed vs predicted bilinear unbiased estimator (BLUEs) in 2 years of study (initiation of booting, *n* = 19) and 3 years of study (7 days after anthesis, *n* = 30). Lines represent the linear regression when statistically significant relationships were found.

**FIGURE 7:**
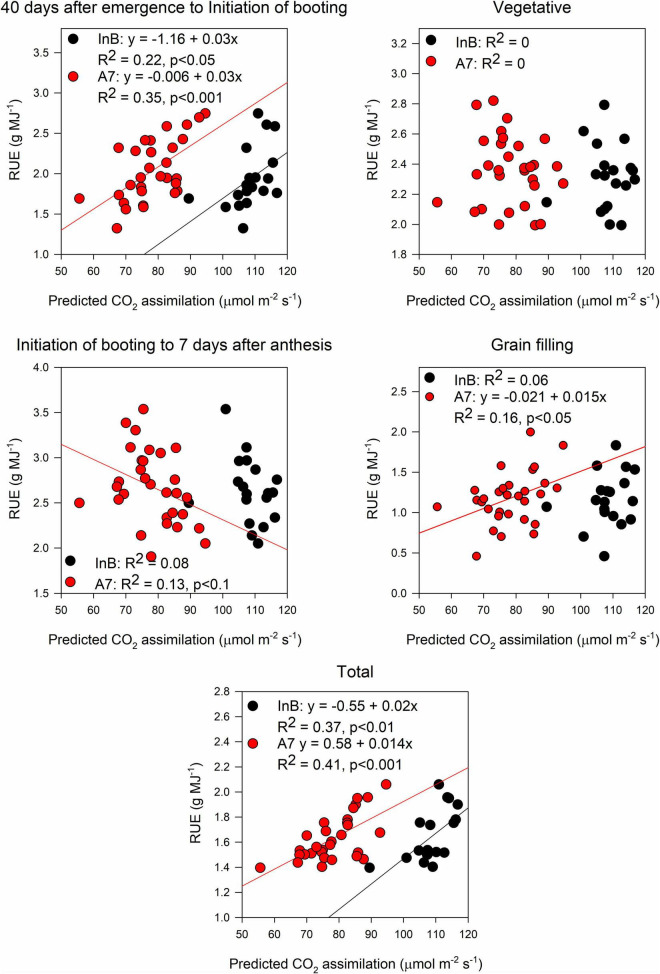
Relationship between radiation use efficiency measured at different growth periods and predictions of canopy assimilation estimated from the PLSR models. Black dots represent predictions from initiation of booting and red dots predictions from 7 days after anthesis. Data shown are the observed vs predicted bilinear unbiased estimator (BLUEs) in 2 years of study (initiation of booting, *n* = 19) and 3 years of study (7 days after anthesis, *n* = 30). Lines represent the linear regression when statistically significant relationships were found.

The prediction accuracy for canopy A_*sat*_ at InB was better (*R*^2^ = 0.3, *p* < 0.05) than predictions at A7 (*R*^2^ = 0.15, *p* < 0.05; Figure 6A). For averaged *gs*, our results showed significant correlations between ground truth data and predictions both at InB (*R*^2^ = 0.29, *p* < 0.05) and A7 (*R*^2^ = 0.65, *p* < 0.001; Figure 6B). The positive correlations between RUE from canopy closure to GS41 (RUE_E40InB) with predicted canopy A_*sat*_ from InB (*R*^2^ = 0.22, *p* < 0.05) and A7 (*R*^2^ = 0.35, *p* < 0.001) were statistically significant. RUE from InB to A7 only correlated marginally significant with the predictions of canopy A_*sat*_ at A7 (*R*^2^ = 0.13, *p* < 0.1). No significant correlations were found for RUE vegetative, and this could be due to the use of A_*sat*_ rates from the middle and bottom of the canopy where light saturation is less prevailing than in the top layer, but correlations between RUE from grain filling and canopy A_*sat*_ A7 were found (*R*^2^ = 0.16, *p* < 0.05). Finally, the correlations found between RUE of the whole crop cycle (RUE_Total) and canopy A_*sat*_ predictions were positive and the strongest of any growth stage (*R*^2^ = 0.37, *p* < 0.01 for InB; and *R*^2^ = 0.41, *p* < 0.001 for A7; Figure 7).

**FIGURE 8:**
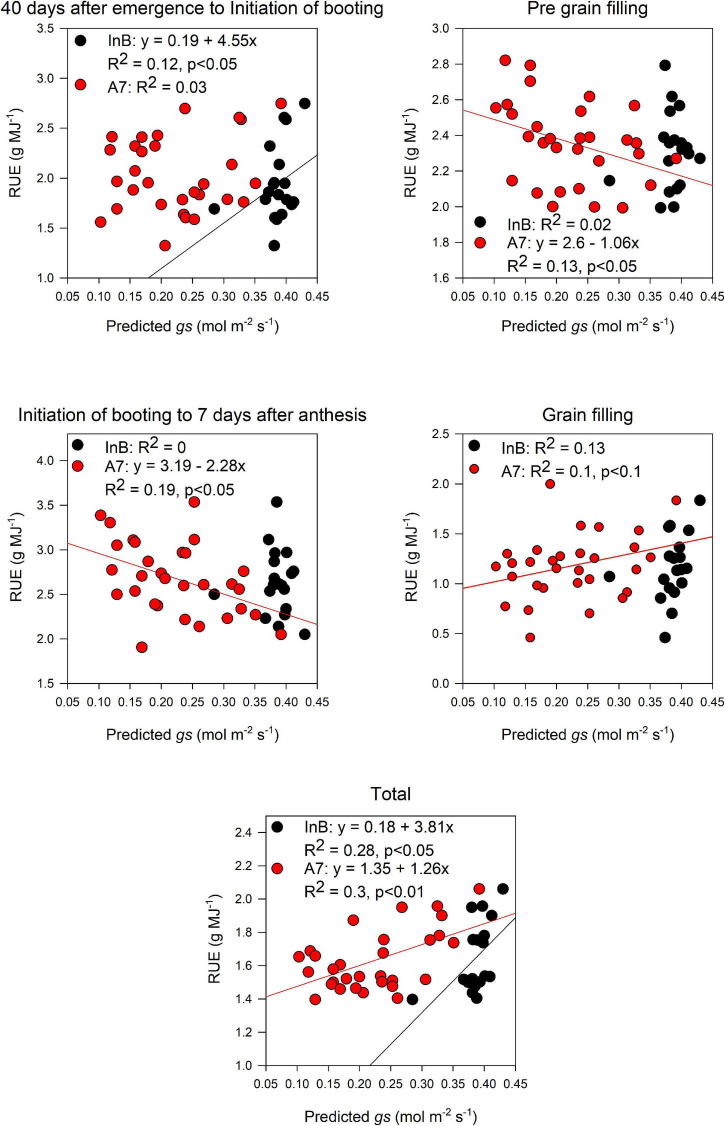
Relationship between radiation use efficiency measured at different growth periods and predictions of stomatal conductance average throughout the canopy estimated from the PLSR models. Black dots represent predictions from initiation of booting and red dots predictions from 7 days after anthesis. Data shown are the observed vs predicted bilinear unbiased estimator (BLUEs) in 2 years of study (initiation of booting, *n* = 19) and 3 years of study (7 days after anthesis, *n* = 30). Lines represent the linear regression when statistically significant relationships were found.

Predicted pooled *gs* at InB correlated significantly with RUE from 40 days after emergence to InB (*R*^2^ = 0.12, *p* < 0.05) and RUE_Total (*R*^2^ = 0.28, *p* < 0.05). For predictions at A7, significant correlations were found with RUE from InB to A7 (*R*^2^ = 0.19, *p* < 0.05), RUE vegetative (*R*^2^ = 0.13, *p* = 0.05), and RUE_Total (*R*^2^ = 0.3, *p* < 0.01). Marginally significant correlations were also found with RUE from grain filling (*R*^2^ = 0.1, *p* < 0.1; Figure 8). Finally, for most of the growth stages where we found relationships between RUE and the predicted canopy traits, these relationships were positive.

## Discussion

Natural variation of photosynthetic traits has not been fully exploited in breeding programs, representing a crucial untapped resource fundamental to increase wheat yields (Molero and Reynolds, 2020). Mainstreaming photosynthetic traits into breeding pipelines has been limited by the lack of methods to quantify them in an HTP context under field conditions.

Leaf and canopy hyperspectral reflectance measurements have largely been acknowledged as proxies with the potential to quantify different photosynthetic, biophysical, and biochemical traits at HTP. Previous studies combining spectral reflectance and PLSR modeling to predict physiological traits have mostly focused on sunlit leaves at the top of the canopy (Silva-Perez et al., 2018; Ely et al., 2019; Fu et al., 2019; Meacham-Hensold et al., 2020), and thus they may not be representative of the whole canopy. In contrast, our models were developed to predict physiological traits within the canopy during the vegetative and grain-filling wheat stages.

Our approach showed that the best predictions were achieved when the three layers of the canopy were combined and compared to using individual layers for the most traits measured. The results in this study add relevance to the measurement of physiological traits not only in the top layer of wheat canopies but highlight middle and bottom layers as they improved the accuracy of the models and can provide robust information to find wheat genotypes that could adapt better to light gradients and exploit them to increase canopy photosynthesis.

The use of the leaf clip attached to the field spectroradiometer allowed us to comfortably perform measurements at the top and middle layers of the canopy, but at the bottom layer, measurements became hard to do. Therefore, new spectroradiometer alternatives that are lighter and easier to deploy in field conditions should be considered to improve bottom layer phenotyping as this is still necessary to measure lower parts of the canopy as UAVs are only able to collect data from the top layer of the canopy.

### Photosynthesis High-Throughput Phenotyping

The prediction accuracy for A_*sat*_ in this study (*R*^2^ = 0.48, RMSE = 5.24) is within the range of previous studies in spring bread wheat (*R*^2^ = 0.49, RMSE = 3.93; Silva-Perez et al., 2018), brassica, moricandia and maize (*R*^2^ = 0.49, RMSE = 4.98; *R*^2^ = 0.37, RMSE = 4.98; *R*^2^ = 0.62, RMSE = 3.64, respectively; Heckmann et al., 2017), and tobacco measured above the canopy (450–900 and 450–1,700 nm) and in the top leaf layer (350–2,500 nm) (*R*^2^ = 0.54, 0.5, 0.56; RMSE = 7.77, 8.52, and 7.04, respectively; Meacham-Hensold et al., 2020). But they were lower compared to reports in tropical trees (*R*^2^ = 0.74, RMSE = 2.85; Doughty et al., 2011) and wheat grown under different salinity concentrations (*R*^2^ = 0.73, RMSE = 2.25; El-Hendawy et al., 2019). Including different layers of the canopy in our models improved the accuracy compared to only predicting the top layer (*R*^2^ = 0.11, RMSE = 4.96, Figure 1). This makes the case of accounting for the variability associated with leaf area, incident radiation levels, and N content in the canopy, which affects light scattering in the canopy and influences A_*sat*_ rates. Furthermore, our models include vegetative and grain filling stages which allows the flexibility of predicting photosynthesis throughout the growth cycle.

The variation of A_*sat*_ within canopy layers can be explained by genetic variation of canopy architecture found in LAI and SLA (Table 2), as light penetrating in areas of the canopy where leaves are smaller (and usually erect) will cause differences in light quality and quantity in the bottom layers of the canopy where large amounts of diffuse radiation and decreased red:far red and blue:red ratios compared to the top layers are found (Burgess et al., 2021).

Stomatal conductance has been predicted previously only in wheat (Silva-Perez et al., 2018; El-Hendawy et al., 2019; Furbank et al., 2021). In spring wheat elite and landrace cultivars were grown in Northwest Mexico, and the prediction accuracies for *gs* were the lowest for a set of traits studied (*R*^2^ = 0.34, RMSE = 0.15) and had the largest associated prediction error (Silva-Perez et al., 2018). In salt-sensitive and tolerant genotypes El-Hendawy et al. (2019) found very high associations between observations and predictions of *gs* between genotypes, growing seasons, and salt tolerance treatments (*R*^2^ = 0.75). Furbank et al. (2021) tested different methods to predict photosynthetic traits, and for *gs*, they found a performance of *R*^2^ = 0.42 in flag leaves using PLSR modeling. In our study, *gs* predictions were weaker than A_*sat*_ predictions (*R*^2^ = 0.36, RMSE = 0.14; Table 1), and assessing this through the different layers of the canopy can help us to understand why that is the case.

Our layer approach shows that there is a higher prediction accuracy in the middle layer of the canopy compared to the top and bottom layers, and this could be explained by the environmental factors affecting *gs*, such as stomatal responses to sunflecks at the top of the canopy, the temperature and vapor pressure deficit differential within the canopy layers, wind speed affecting the boundary layer especially at the top, relative humidity, leaf water content, and CO_2_ depletion in sunny days. Hence, the lack of studies predicting *gs* under field conditions and future studies should consider the influence of the abovementioned environmental factors when building predictive models, a combination between PLSR and thermography, or the use of deep learning methods (Supplementary Figure 1; Furbank et al., 2021).

Chlorophyll content has been used as an important trait to assess photosynthetic capacity, the ability of canopies to intercept light, and the time wheat can maintain photosynthetically active tissues during the crop cycle. SPAD measurements have become one of the standard proxies to estimate chlorophyll content in the field. Our predictions for SPAD values were lower than the ones reported in a previous study measuring elite and landrace bread wheat cultivars growing under yield potential conditions in the same study site (*R*^2^ = 0.63 vs *R*^2^ = 0.82, in flag leaves; Supplementary Figure 2; Silva-Perez et al., 2018; Furbank et al., 2021). In general, the predictions of chlorophyll content ranked very high in terms of accuracy (*R*^2^, RMSE) in this study compared to other traits, a similar trend found for tobacco (Meacham-Hensold et al., 2020) and tropical tree species (Doughty et al., 2011).

### Speeding Up Physiological Breeding

The use of HTP methods for physiological breeding has increased in popularity, particularly the use of field spectroradiometers, hyperspectral cameras mounted on UAVs, or modified IRGAs that are deployed in glasshouses and field trials in conjunction with commercial IRGAs. The use of these technologies can reduce dramatically the measurement time, for example, for A_*sat*_ measurements take ∼15–25 min per leaf using a commercial IRGA compared to 1 min when collecting leaf spectral data (Heckmann et al., 2017). The use of HTP in this study allowed us to screen ∼50 plots for flag, second, and third leaves reflectance in ∼1 h compared to only 10 plots using two commercial IRGAs during 6 h of field measurements in a day, thus increasing our phenotyping capacity 30-fold. Coupling approaches like the one used in our study based on hyperspectral data combined with the modeling of performance physiological traits, such as biomass and RUE (Robles-Zazueta et al., 2021), can boost the phenotyping capacity in large breeding trials, increase our understanding of the source-sink relationship, and help with the selection of genotypes with higher biomass, RUE, and yield.

The relationship found between canopy assimilation predictions and RUE observations could be used for screening RUE in breeding programs and can be coupled with the previous results from Robles-Zazueta et al. (2021). RUE could be screened with up to 70% accuracy using vegetation indices, and the predictions presented in this study could be used to screen lines for extreme high and low RUE rates as the positive relationship found between RUE and canopy assimilation predictions indicate that the higher predicted values are we expect to screen genotypes with higher RUE.

Furthermore, the next steps that should be taken are to compare the prediction vs. ground truth data heritability to make the case to incorporate these predictions in breeding programs and define what is the minimum genotypic, environmental variation, and sample size needed to build accurate models of complex traits. Previous studies show an extreme range variation with models built from 50 samples (Heckmann et al., 2017) to as large as 2,478 data points (Serbin et al., 2019). It seems that the degree of accuracy of each trait modeled with hyperspectral reflectance increases when combining different plant species rather than working with different genotypes of the same crops (e.g., wheat or maize), and this could be the result of the stress responses of diverse plant communities compared to the responses of a variety of genotypes from the same species growing in controlled environments (Grzybowski et al., 2021) or building models with environmental conditions that might not be replicated the following year, thus affecting prediction results.

For these reasons, we suggest building the models with data from multiple field cycles (at least two) to create models which can predict desired traits in a range of growing environmental conditions for wheat, although in our work two field cycles were not accurate, perhaps for the small number of lines studied (Supplementary Figure 3) as opposed to three field cycles which were more accurate due to the addition of year by year variability and a larger sample size (Figure 4). Additionally, new technologies should be deployed in the field to allow the assessment of hyperspectral reflectance in all plant organs including ears (Vergara-Diaz et al., 2020), stems, and if possible, all the different leaves within the canopy to have a better picture of wheat physiological processes while assuring that the predictions have relevance for breeders.

The importance of VIP scores relies on identifying the wavelengths from the electromagnetic spectrum with higher predictive power. In this study, three relevant areas of the spectra were found to have greater importance to build the models for all the traits measured. Those peaks were found at 350–369, 527–575, and 671–750 nm (Figure 5). The first two areas are located in the visible region, which is an area related to pigment content, such as anthocyanins, carotenoids, xanthophylls, chlorophyll a and b, as well as light interception traits, such as canopy greenness, LAI, photosynthetic capacity (A_*sat*_, A_*max*_), NPQ (Malenovský et al., 2009; Gamon et al., 2019), and light use efficiency (LUE; Blackburn, 2007). The third peak was found in the region known as “red edge,” which is related to canopy greenness, chlorophyll content, chlorophyll fluorescence, and solar-induced fluorescence (SIF), which has gained recent attention for its potential use as a proxy to measure photosynthesis in crops (Pinto et al., 2020). With our VIP scores, models for functional traits could be made to simplify data management by only using the wavelengths with greater predictive ability.

## Concluding Remarks

This is the first study where physiological traits in the top, middle, and bottom layers of wheat canopies were predicted by building models with hyperspectral data using PLSR. We showed that integrating measurements from the different canopy layers improved the accuracy of the models in most traits studied. These models can be used to study the variation caused by different environmental conditions within the canopy and the effect of phenology. Our models were built using an extensive dataset from three field campaigns, which provides them robustness, enabling their application in future field trials. Furthermore, this modeling approach delivered fair estimations of A_*sat*_ and *gs* that can be incorporated in breeding pipelines. Using hyperspectral data will allow the alleviation of the phenotyping bottleneck, and if this approach is coupled to faster phenotyping platforms the probabilities to screen genotypes for higher photosynthesis and RUE will increase.

## Data Availability Statement

The raw data supporting the conclusions of this article will be made available by the authors, without undue reservation.

## Author Contributions

CAR-Z, FP, GM, MPR, and EHM conceived the original research plan. CAR-Z, FP, and GM designed the field experiment. CAR-Z performed the field experiments, collected and analyzed the data with supervision of FP, GM, MJF, MPR, and EHM, and wrote the manuscript with contributions from co-authors. FP, GM, MPR, and EHM obtained project funding. MPR and EHM managed project funding. All authors contributed to the article and approved the submitted version.

## Conflict of Interest

The authors declare that the research was conducted in the absence of any commercial or financial relationships that could be construed as a potential conflict of interest.

## Publisher’s Note

All claims expressed in this article are solely those of the authors and do not necessarily represent those of their affiliated organizations, or those of the publisher, the editors and the reviewers. Any product that may be evaluated in this article, or claim that may be made by its manufacturer, is not guaranteed or endorsed by the publisher.

## Supplementary Material

Supplementary Figure 1 Observations of A_*sat*_ and *gs* compared to the predictions from the models developed in Furbank et al. (2021). Measurements from flag leaves were compared to predictions made with flag leaf reflectance from the vegetative and grain-filling periods. The modeling was done using the web tool Wheat Physiology Predictor (https://plantpredict.shinyapps.io/PredictionShiny/). We selected the different methods available in the tool to compare the prediction ability. Methods included single task convolutional neural network (black dots), multi-task convolutional neural network (red dots), partial least square regression (blue dots), and an ensemble of the three models (green dots). Lines represent the linear regression when relationships between ground truth data and predictions were statistically significant.

Supplementary Figure 2 Observations of LMA and SPAD compared to the predictions from the models developed in Furbank et al. (2021). Measurements from flag leaves were compared to predictions made with flag leaf reflectance from the vegetative and grain-filling periods. The modeling was done using the web tool Wheat Physiology Predictor (https://plantpredict.shinyapps.io/PredictionShiny/). We selected the different methods available in the tool to compare the prediction ability. Methods included single task convolutional neural network (black dots), multi-task convolutional neural network (red dots), partial least square regression (blue dots), and an ensemble of the three models (green dots). Lines represent the linear regression when relationships between ground truth data and predictions were statistically significant.

Supplementary Figure 3 Predictions with models built with a dataset from Y1 and Y2 to estimate data from Y3 of A_*sat*_ **(A)** and *gs* **(B)** by separating each layer of the canopy (top panels) and predictions of A_*sat*_ **(C)** and *gs* **(D)** combining all the layers of the canopy (black squares). Black dots: top of the canopy, blue dots: middle of the canopy, gray dots: bottom of the canopy. The lines represent the linear regression between predictions and ground truth data.
