# Supplementary figures and images for: Prediction of Photosynthetic, Biophysical, and Biochemical Traits in Wheat Canopies to Reduce the Phenotyping Bottleneck
Source: Front Plant Sci. 2022 Apr 11;13:828451. doi: 10.3389/fpls.2022.828451 (PMC9036448; doi:10.3389/fpls.2022.828451)

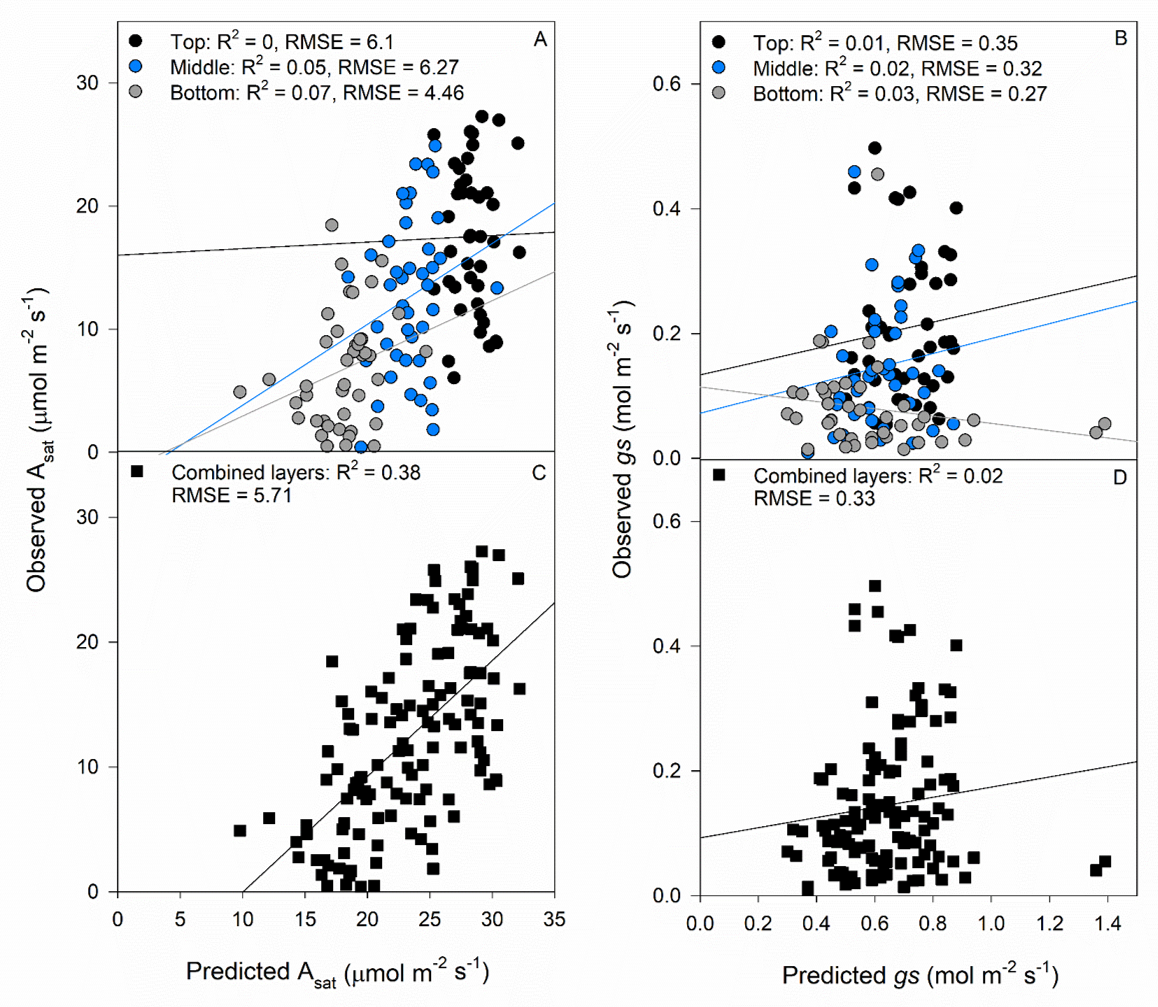

Supplement: Supplementary Figure 3 — Predictions with models built with a dataset from Y1 and Y2 to estimate data from Y3 of Asat (A) and gs (B) by separating each layer of the canopy (top panels) and predictions of Asat (C) and gs (D) combining all the layers of the canopy (black squares). Black dots: top of the canopy, blue dots: middle of the canopy, gray dots: bottom of the canopy. The lines represent the linear regression between predictions and ground truth data. [file Image_3.TIF]
